# Impact of thyroid hormones on predicting the occurrence of persistent inflammation, immunosuppression, and catabolism syndrome in patients with sepsis

**DOI:** 10.3389/fendo.2024.1417846

**Published:** 2024-10-16

**Authors:** Ling Zhang, Ruoming Tan, Tingting Pan, Hongping Qu

**Affiliations:** Department of Critical Care Medicine, Ruijin Hospital, Shanghai Jiao Tong University School of Medicine, Shanghai, China

**Keywords:** sepsis, critical care, PICs, chronic critical illness, thyroid hormone

## Abstract

**Background:**

The prevalence of persistent inflammation, immunosuppression, and catabolism syndrome (PICS) has an upward trend in sepsis patients and can be associated with poor outcomes. Thyroid hormones are expected to be correlated with inflammation, immunity, and metabolism. Thus, the purpose of this study was to evaluate the effect of thyroid hormones on the occurrence of PICS and then further explore the optimal level of them in sepsis.

**Methods:**

This retrospective observational study used the online database Medical Information Mart for Intensive Care (MIMIC)-IV. Univariate and multivariate logistic regression analyses were employed to determine correlations between thyroid hormone levels and PICS. A combination of independent PICS development factors was established with accuracy assessed using the area under the receiver operating characteristic curve (AUC-ROC).

**Results:**

Patients were divided into PICS (n=205) and non-PICS (n=671) groups. The third quartiles of triiodothyronine (T3) (60-80ng/dl) and thyroxine (T4) (5.5-6.8ug/dl) had the lowest PICS incidence and the adjusted odds ratio (OR) was 0.33 (T3, p=0.009) and 0.39 (T4, p=0.006), respectively, compared with the first quartiles of T3 and T4. For patients with a pre-existing T3 deficiency, severe deficiency (T3 <60ng/dl) and a high Sequential Organ Failure Assessment (SOFA) score were significantly related to PICS incidence. The AUC for these combined parameters in predicting PICS occurrence was 0.748 (all patients) and 0.861 (patients without thyroid disease).

**Conclusions:**

A mild T3 deficiency (60-80ng/dl) was significantly associated with the lowest risk-adjusted PICS occurrence in patients with sepsis. A severe T3 deficiency (<60ng/dl) and a high SOFA score were independent risk factors for PICS occurrence.

## Background

Sepsis affects millions of people worldwide annually and severe infection is associated with high mortality (1, 2). Due to the progress of intensive care therapy, hospital mortality caused by sepsis has declined (3). However, this has resulted in an increase in chronic critical illness (CCI) patients with sustained impairment of cognition and organ function and relatively poor long-term survival (4). A proportion of CCI patients may develop persistent inflammation, immunosuppression, and catabolism syndrome (PICS) with persistent inflammation [e.g., high C-reactive protein (CRP)], immunosuppression (e.g., lymphocytopenia), and catabolism (e.g., low albumin) (5–7). PICS is related to longer length of stay in hospital, higher mortality, and lower long-term quality of life (8). Therefore, it is important to identify clinical characteristics to accurately predict PICS occurrence and develop preventative strategies.

Although the mechanisms underlying PICS remain unclear, increasing evidence shows that altered myelopoiesis with expanded myeloid-derived suppressor cells, reduced function of effector T cells, and protein catabolism caused by malnutrition are the dominant contributing factors (9, 10). In addition to changes in inflammatory, immune, and catabolic indicators, CCI is associated with endocrine dysfunction, including the suppression of pulsatile thyroid stimulating hormone (TSH), thyroid hormones (THs), growth hormone, prolactin, and insulin growth factor-I (11, 12). However, few studies have explored the influence of endocrine hormones on the occurrence of PICS in patients with sepsis.

As important endocrine hormones, THs, and especially triiodothyronine (T3), have direct effects on the utilization of adenosine triphosphate, causing the acceleration of macronutrient metabolism (e.g., protein catabolism and fatty acid oxidation) (13). The influence of thyroid function on innate and adaptive immune responses is also supported by clinical and preclinical studies, mediated by multiple pathways (14, 15). Furthermore, in critically ill patients, and especially in those with sepsis, the hypothalamic-pituitary-thyroid (HPT) axis is always significantly disturbed (16), for instance, there may be reduced T3 or free T3 (FT3) and elevated reverse T3 (rT3), which is converted by thyroxine (T4) via inner ring deiodination. This phenomenon can be partly explained by a physiological adaptation to reduce energy expenditure to limit catabolism (17, 18), and appears to be correlated with poor outcomes, including higher mortality and hospitalization expenses (19–21).

Considering the bidirectional relationship between THs and inflammation, immunity, and metabolism, which in turn may influence thyroid function, we hypothesized that THs, particularly T3, may play a crucial role in the occurrence of PICS. Thus, this study aimed to examine the impact of THs on PICS development in patients with sepsis and further explore the appropriate levels of THs.

## Methods

### Data source

This retrospective observational study used the Medical Information Mart for Intensive Care (MIMIC)-IV (version 1.0) database (22), which was developed and maintained by the Laboratory for Computational Physiology at the Massachusetts Institute of Technology (MIT). The MIMIC-IV database integrated comprehensive clinical information from patients in the intensive care unit (ICU) of the Beth Israel Deaconess Medical Center in Boston, Massachusetts, USA, from between 2008 and 2019. One author, Ling Zhang, passed the Collaborative Institutional Training Initiative Examination and obtained permission to extract data (certification number:47869408).

### Patient population and variable extraction

Our study included 35,010 patients diagnosed with sepsis based on the MIMIC-IV database. Sepsis was identified as patients with documented or suspected infection and an acute change of ≥2 points in their Sequential Organ Failure Assessment (SOFA) score, according to the Sepsis 3.0 guidelines (23). The inclusion criteria were: (1) age ≥18 years; (2) first time in ICU; (3) time in ICU ≥48 h; and (4) measurement of at least one TH (T3, T4, or FT4) within 24 h before and 7 d after ICU admission. Pregnant or lactating women were excluded.

After removing patients who had died within 10 days after ICU admission, the remaining patients were divided into the PICS group (defined as CRP >3.0 mg/dL, lymphocyte count <800/μL, or albumin <3.0 g/dL on 1 day 10 days after ICU admission) and the non-PICS group (defined as CRP, lymphocyte count, and albumin were all negative or discharged alive within 10 days after ICU admission) (24, 25).

### Variable extraction

The following variables were extracted from the MIMIC-IV database:

1. Patient characteristics: age, gender, weight, body mass index (BMI);
2. Clinical Severity scores: SOFA score and Simplified Acute Physiology Score II (SAPS II) score;
3. Comorbidities: Charlson comorbidity score, thyroid disease (including hypothyroidism, hyperthyroidism, thyrotoxicosis, and thyroiditis), and immune deficiencies [including HIV/AIDS, lymphoma, metastatic cancer, transplantation, and autoimmune disease (26)], defined according to the ICD-9 or ICD-10 diagnosis codes;
4. Laboratory variables: white blood cell count, neutrophil count, lymphocyte count, hemoglobin, platelets, lactate, creatinine, urea, albumin, and aspartate aminotransferase (AST) within 24 h after ICU admission; blood cortisol within 72 h after ICU admission; and THs within 24 h before and 7 d after ICU admission;
5. Interventions: use of mechanical ventilation, renal replacement therapy (RRT), vasopressors in the first 24 h, and corticosteroid use, including dexamethasone, hydrocortisone, and methylprednisolone within 10 d after ICU admission.

### Definitions and outcomes

Non-thyroidal illness syndrome (NTIS) was defined as a T3 level below the normal range (80-200 ng/dl), with thyroid-stimulating hormone (TSH) not increasing, during a critical illness (27). The primary outcome was PICS incidence. The data were further analyzed for length of stay and mortality in ICU and hospital, 28-day mortality, and discharge destination.

### Statistical analysis

The continuous variables were presented as mean ± standard deviation and compared using Student’s t-test when the data were normally distributed. Non-normally distributed continuous variables were presented as median (interquartile range) and compared using the nonparametric Mann–Whitney U or Kruskal–Wallis tests. The categorical variables were expressed as proportions and compared using the chi-squared test. The variables with statistical significance in the univariate logistic regression analysis or with clinical relevance were included in a multivariate analysis to ascertain the independent risk factors for the occurrence of PICS. The diagnostic accuracy for the incidence of PICS was evaluated using area under the receiver operating characteristic curve (AUC-ROC) analysis. Subgroup analysis according to baseline thyroid disease status was performed.

A two-sided analysis with a p-value < 0.05 was considered statistically significant. Data were analyzed using IBM SPSS Statistics version 25.0 and R language version 3.6.1.

## Results

### Population and baseline characteristics

**Figure 1:**
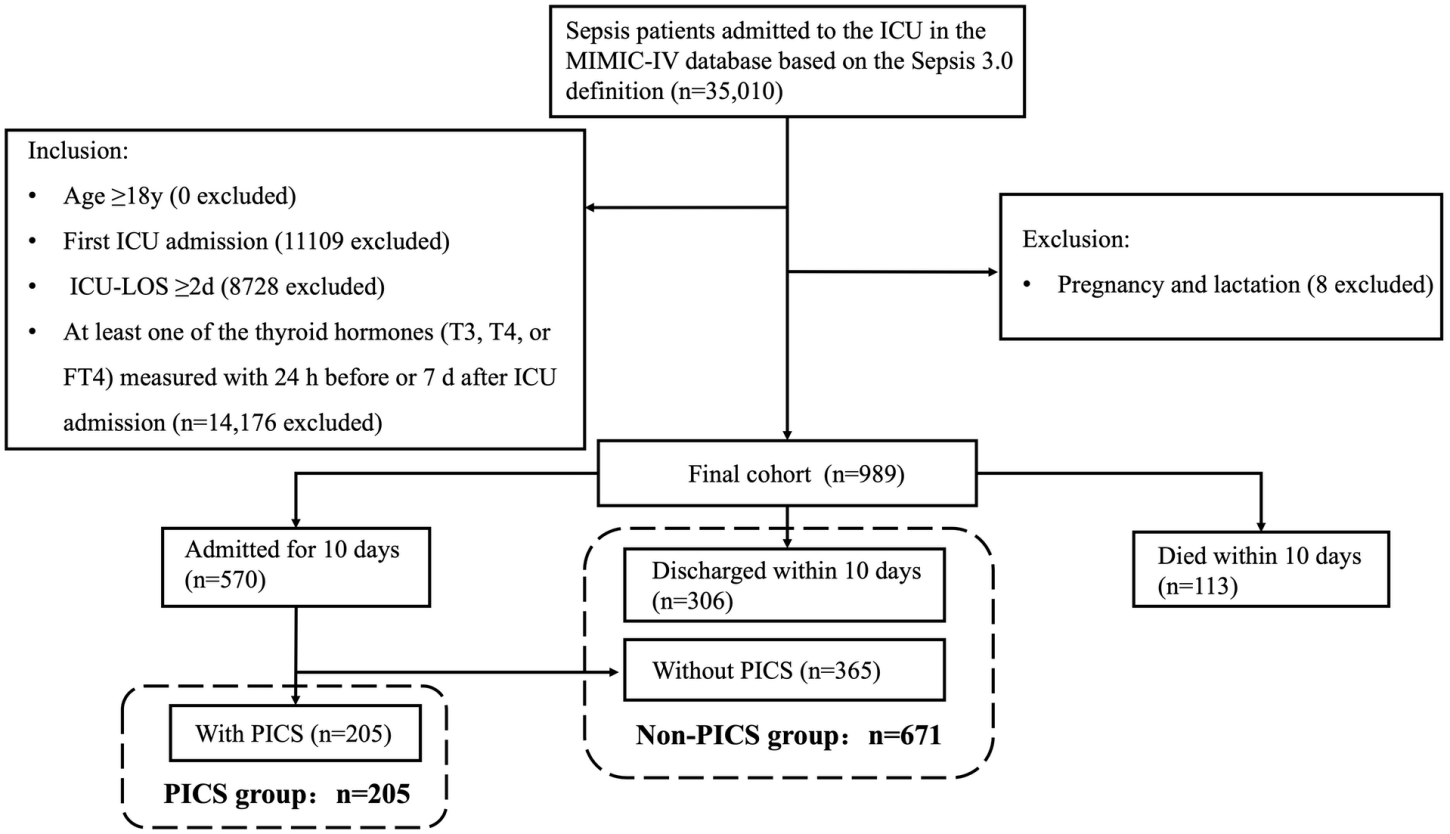
Flowchart of the study participants. LOS, length of stay; PICS, persistent inflammation, immunosuppression, and catabolism syndrome.

The study population flow chart is shown in **Figure 1**. In the MIMIC-IV database, 35,010 ICU admissions fulfilled the criteria for sepsis. Following the application of the inclusion and exclusion criteria, 989 eligible patients were enrolled in the final cohort. Within the first 10 d after ICU admission, 113 patients died and 306 were discharged. Of the remaining 570 patients, 205 had CRP >3.0 mg/dL or a lymphocyte count <800/μL or albumin <3.0 g/dL 10 d after ICU admission (PICS group) and 365 patients did not develop PICS. The non-PICS group (671 patients) included the 365 patients without PICS and the 306 discharged patients.

**Table 1** presents the baseline characteristics for the PICS and non-PICS groups. The groups were similar in age, gender, weight, and BMI; however, the patients in the PICS group were more critically ill with worse initial laboratory data and more frequent use of mechanical ventilation, renal replacement therapy, and vasopressors (p <0.05). Moreover, compared with the non-PICS group, the PICS group had lower thyroid disease morbidity (33.7% vs. 43.1%) and higher immune deficiency-related morbidity (21.5% vs. 15.4%). By definition, 51.4% and 38.9% of patients in PICS and non-PICS group, respectively, were identified as having NTIS (p=0.071).

**Table 1:** Baseline characteristics and comparisons between PICS and non-PICS .

Characteristic	PICS (n=205)	Non-PICS (n=671)	P
**Age (years)**	66.0 (54.6,77.5)	68.5 (56.0,79.3)	0.122
**Male, n (%)**	101 (49.3)	309 (46.1)	0.419
**Weight (kg)**	74.4 (63.9,96.0)	77.8 (64.8,94.0)	0.434
**BMI (kg/m^2^)**	27.26 (23.35,32.43)	28.14 (24.04,33.38)	0.134
**SAPS II score**	44 (26,54.5)	39 (31,49)	<0.001
**SOFA score**	9 (6,12)	6 (4,9)	<0.001
Comorbidities			
Charlson comorbidity score	6 (4,8)	6 (4,8)	0.060
Thyroid disease, n (%)	69 (33.7)	289 (43.1)	0.016
Immunocompromised disease, n (%)	44 (21.5)	103 (15.4)	0.040
Laboratory findings			
WBC (×10^9^/L)	13.65 (8.43,19.20)	13.00 (9.25,17.65)	0.909
Neutrophil count (×10^9^/L)	9.69 (5.68,16.60)	9.59 (5.93,17.19)	0.393
Lymphocyte count (×10^9^/L)	0.82 (0.47,1.24)	0.99 (0.59,1.53)	0.007
Neutrophil: lymphocyte	11.42 (6.76,20.43)	9.59 (5.93,17.19)	0.114
Hemoglobin (g/dl)	9.5 (7.9,11.4)	10.0 (8.6,11.6)	0.009
Platelets (×10^9^/L)	147 (90,206)	180 (123,245)	<0.001
CRP (mg/L)	112.0 (57.0,174.7)	77.4 (26.3,132.7)	0.102
Lactate (mmol/L)	2.3 (1.6,4.4)	2.0 (1.4,3.6)	0.009
Urea (mg/dl)	9.64 (6.25,16.42)	8.93 (5.71,15.35)	0.081
Creatinine (mg/dl)	114.92 (79.56,207.74)	114.92 (70.72,185.64)	0.559
Urea: creatinine	78.59 (54.88,120.63)	75.25 (56.63,103.85)	0.177
Albumin (g/dl)	2.8 (2.4,3.4)	3.2 (2.7,3.6)	<0.001
AST (IU/L)	64 (35,174)	42 (26,116)	0.001
NTIS (%)	36 (51.4)	74 (38.9)	0.071
Cortisol (ug/dl)	21.6 (13.0,32.6)	20.5 (11.4,29.6)	0.338
Interventions			
RRT use, n (%)	25 (12.2)	42 (6.3)	0.005
Ventilation use, n (%)	130 (63.4)	327 (48.7)	<0.001
Vasopressor use, n (%)	116 (56.6)	297 (44.3)	0.002
Corticosteroid use, n (%)	75 (36.6)	158 (23.5)	<0.001

Data are presented as mean ± SD or median (interquartile range) for skewed variables or proportions for categorical variables, respectively.

PICS, persistent inflammation, immunosuppression, and catabolism syndrome; BMI, body mass index; SAPS II, Simplified Acute Physiology Score II; SOFA, Sequential Organ Failure Assessment; WBC, white blood cell; CRP, C-reactive protein; AST, aspartate transaminase; NTIS, non-thyroidal illness syndrome; RRT, renal replacement therapy.

### Outcomes in patients with or without persistent inflammation, immunosuppression, and catabolism syndrome

Compared with the non-PICS patients, patients with PICS had higher ICU mortality (8.8% vs 2.4%), hospital mortality (19.5% vs 5.1%), and 28-day mortality (14.6% vs 5.4%), and had longer ICU (11.4 vs 4.6 days) and hospital stays (26.2 vs 11.1 days). The ratio of patients with PICS to discharge and returning home was also lower (p< 0.001) (see **Supplementary Table S1**).

### Association between thyroid hormone levels and persistent inflammation, immunosuppression, and catabolism syndrome

**Figure 2:**
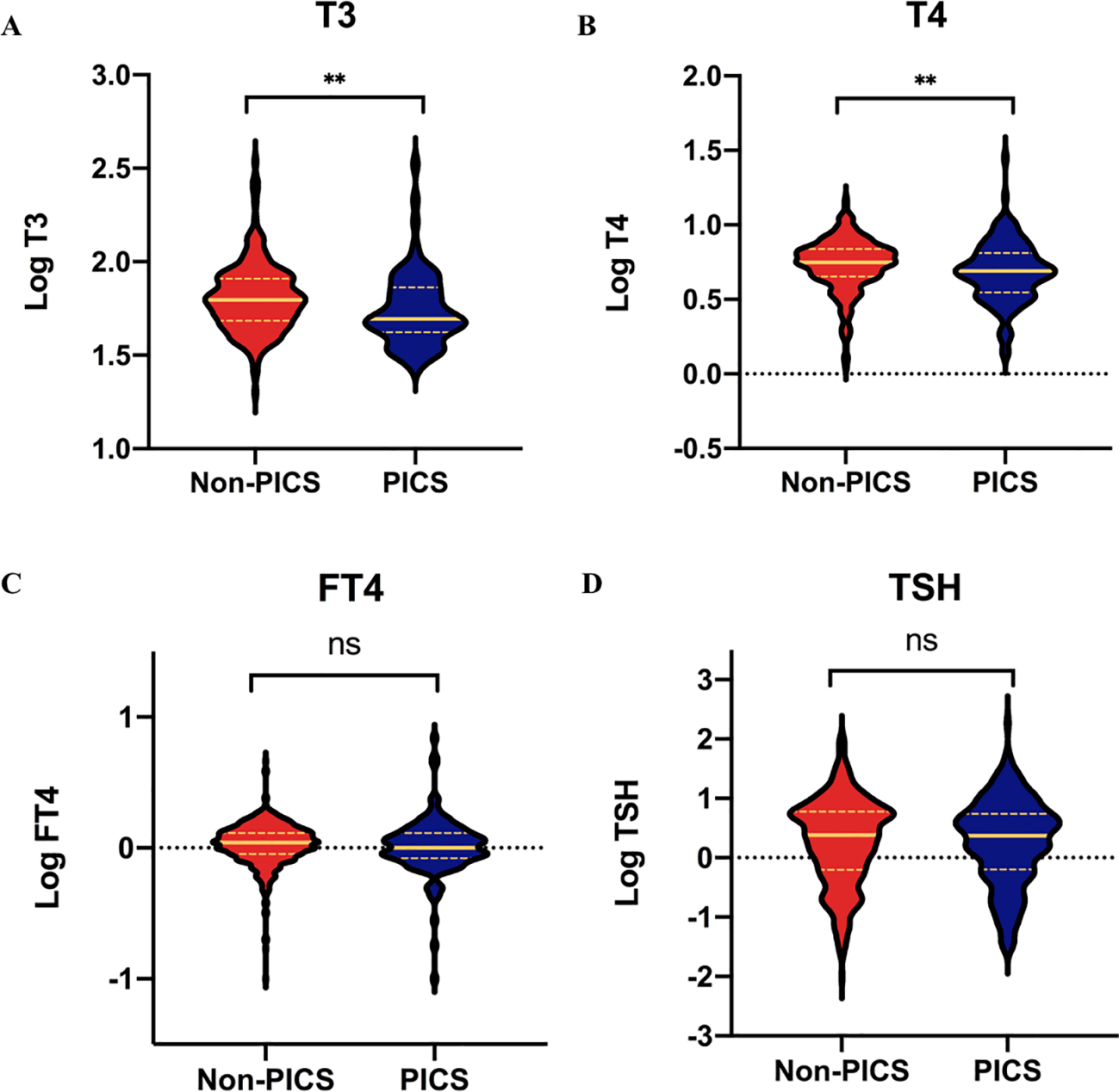
Comparisons of thyroid hormones between the PICS and non-PICS groups. **(A)** T3, **(B)** T4, **(C)** FT4, and **(D)** TSH. ** indicates a significant p-value less than 0.01; ns, no significance; PICS, persistent inflammation, immunosuppression, and catabolism syndrome.

As shown in **Figure 2**, both T3 (49.5 vs 62.5 ng/dl, p=0.003) and T4 (4.9 vs 5.6 ug/dl, p = 0.003) levels were significantly lower in the PICS group compared with the non-PICS group; however, there was no significant difference in TSH and FT4 levels between the two groups. We divided T3 and T4 into quartiles to further analyze their correlation with the development of PICS.

**Figure 3:**
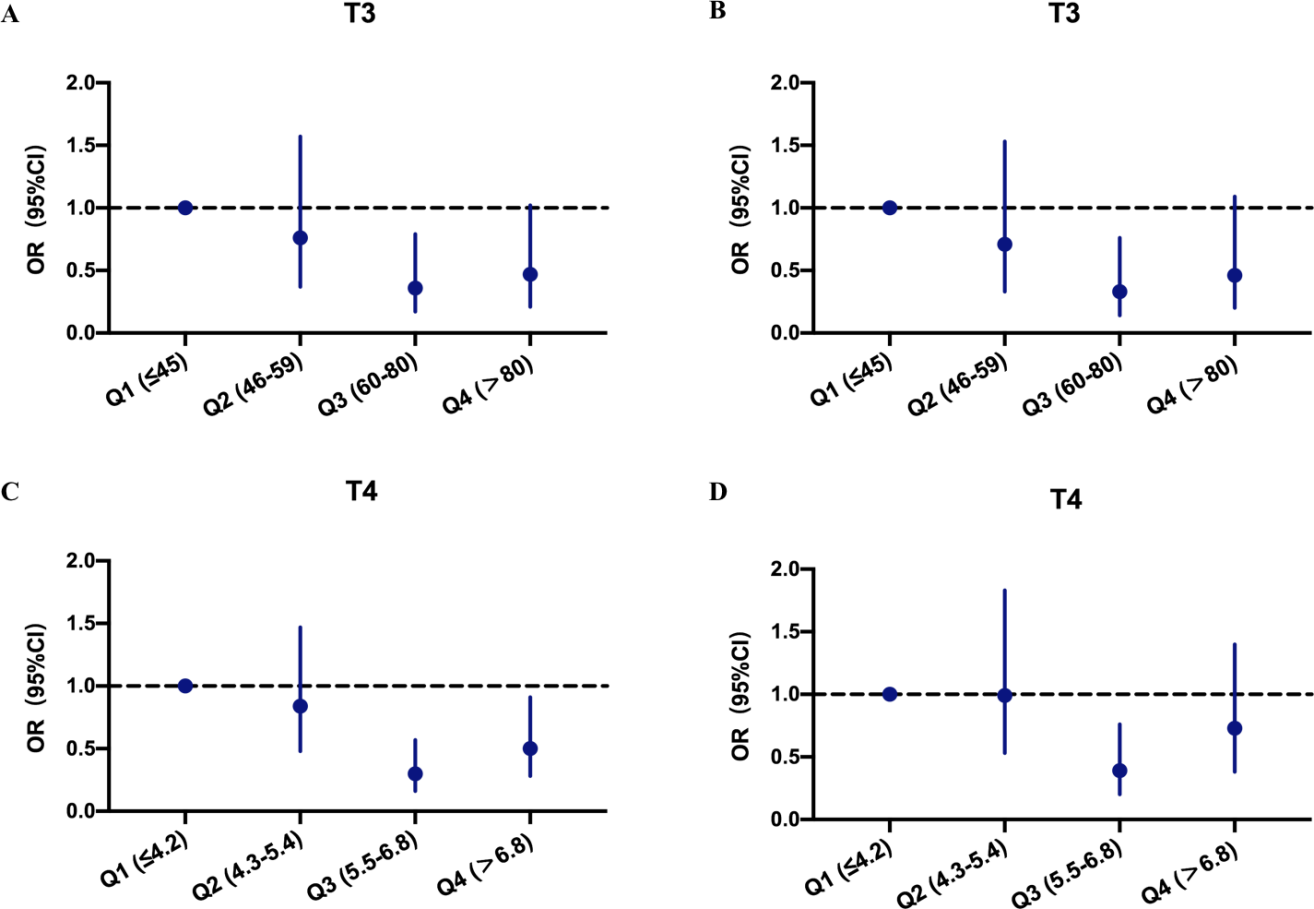
Odds ratios and confidence intervals of PICS occurrence at different thyroid hormone levels. **(A)** Crude analysis at different T3 levels. **(B)** Adjusted analysis for age, gender, SAPS II, SOFA, Charlson comorbidity score, and thyroid disease at different T3 levels. **(C)** Crude analysis at different T4 levels. **(D)** Adjusted analysis for age, gender, SAPS II, SOFA, Charlson comorbidity score, and thyroid disease at different T4 levels. OR, odds ratio; CI, confidence interval; PICS, persistent inflammation, immunosuppression, and catabolism syndrome.

**Figure 3** shows that the third quartiles of T3 and T4 were correlated with the lowest incidence of PICS. The crude odds ratio (OR) for PICS was 0.36 for T3 (**Figure 3A**) and 0.30 for T4 (**Figure 3C**) in the third quartiles compared with the reference group (first quartiles). After adjusting for potential confounders (age, gender, SOFA score, SAPS II score, Charlson comorbidity score, and baseline thyroid disease status), the association between the THs and PICS occurrence remained significant, as the adjusted OR for the third quartile patients was 0.33 for T3 (**Figure 3B**) and 0.39 for T4 (**Figure 3D**).

We then defined the third quartile patients of T3 and T4 as mild T3 deficiency (MD- T3) and mild T4 deficiency (MD-T4), respectively, and the first and second quartile patients as severe T3 deficiency (SD-T3) and severe T4 deficiency (SD-T4), respectively. The patients in the MD-T3 and MD-T4 groups had a significantly lower incidence of PICS; however, in the subgroup with baseline thyroid disease, no correlation between T3 value and PICS incidence was observed (see **Supplementary Table S2**).

### Predictive value of thyroid hormones for persistent inflammation, immunosuppression, and catabolism syndrome

Univariate and multivariate logistic regression analyses were performed to evaluate the baseline risk factors for PICS. According to the univariate regression analysis, we found PICS occurrence was significantly correlated with SOFA score, SAPS II score, hemoglobin, platelets, lactate, AST, baseline thyroid disease, immunocompromised status, and initial T3 concentration in the SD-T3 group. Subsequently, we identified some significant and important factors and analyzed these characteristics using multivariate regression analysis in the patients with an initial T3 deficiency. Finally, SOFA score and SD-T3 group were identified as independent risk factors for PICS (**Table 2**); similar results were observed in patients without initial thyroid disease (see **Supplementary Table S3**). However, we found no association between the initial T4 concentration and PICS development after multivariate regression analysis in the patients with a T4 deficiency.

**Table 2:** Univariate and multivariate logistic regression analysis of PICS in all patients.

Variable	Univariate logistic regression analysis		Multivariate logistic regression analysis	
	Odds ratio (95% CI)	P	Odds ratio (95% CI)	P
Female sex	0.879 (0.643-1.202)	0.419		
Age	0.994 (0.985-1.004)	0.234		
BMI	0.981 (0.958-1.005)	0.120		
SOFA score	1.179 (1.130-1.231)	<0.001	1.379 (1.122-1.695)	0.002
SAPS II score	1.026 (1.014-1.037)	<0.001	0.990 (0.950-1.032)	0.641
Charlson comorbidity score	1.047 (0.992-1.104)	0.096		
SD-T3	2.415 (1.200-4.862)	0.014	3.628 (1.111-11.851)	0.033
T4	0.931 (0.845-1.024)	0.141	0.972 (0.729-1.295)	0.845
WBC	0.992 (0.974-1.010)	0.382		
CRP	1.006 (0.999-1.012)	0.074		
Hemoglobin	0.911(0.847-0.979)	0.011	1.186 (0.948-1.483)	0.136
Platelets	0.997 (0.995-0.999)	<0.001	1.004 (0.999-1.010)	0.094
Lactate	1.098 (1.038-1.162)	0.001	0.958 (0.826-1.111)	0.569
AST	1.000 (1.000-1.000)	0.009	1.000 (0.999-1.000)	0.589
Creatinine	1.000 (0.999-1.001)	0.982		
Thyroid disease	0.671 (0.483-0.930)	0.017	0.428 (0.163-1.123)	0.085
Immunocompromised disease	1.507 (1.016-2.235)	0.041	1.563 (0.466-5.239)	0.469

CI, confidence interval; BMI, body mass index; SOFA, Sequential Organ Failure Assessment; SAPS II, Simplified Acute Physiology Score II; SD-T3, severe deficiency of T3; WBC, white blood cell; CRP, C-reactive protein; AST, aspartate transaminase.

**Figure 4:**
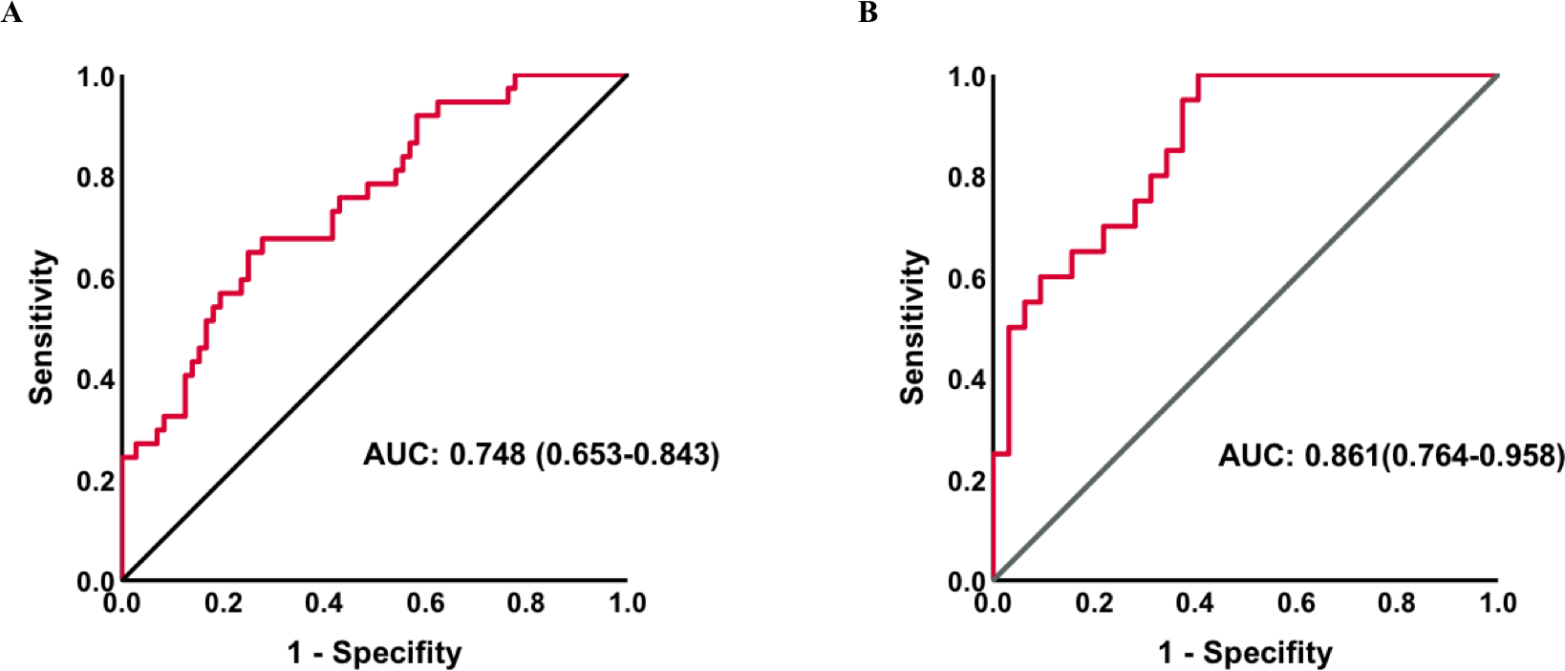
Receiver operating characteristic curves for PICS in all patients **(A)** and a subgroup without thyroid disease **(B)**. AUC, area under the curve; PICS, persistent inflammation, immunosuppression, and catabolism syndrome.

To determine the predictive accuracy, we employed ROC curve analysis. The AUC-ROC of the combination of parameters mentioned in the multivariate regression to predict PICS was 0.748 (95%CI:0.653–0.843) in all patients and 0.861 (95% CI:0.764–0.958) in the subgroup without initial thyroid disease (**Figure 4**).

## Discussion

With the dysregulated response to infection, a clinical manifestation including life-threatening organ dysfunction occurs, named sepsis. Multiple systemic homeostases could be disturbed by it in both the initial acute and chronic stages. For the first time, we demonstrated that both T3 and T4 levels were significantly lower in the PICS group than in non-PICS group, and patients with sepsis with a mild TH deficiency had the lowest PICS incidence. For patients with an initial T3 deficiency, the SD-T3 group and SOFA score were determined to be independent risk factors for PICS, and the combined model of parameters was a good predictor for PICS. Similar results were observed in the subgroup without initial thyroid disease but not in the patients with thyroid disease.

Patients in ICU generally present with a change in the HPT axis with decreased T3, increased rT3, low or low-normal T4, and a lack of a rise in TSH (27). A clinical trial found that 44.1% of ICU patients had reduced FT3 values, and nearly half of these patients had an associated decline in TSH levels, while the other half had normal TSH levels (28). The pathogenesis of these changes in THs may include HPT axis feedback regulation, local thyroid hormone metabolic abnormalities, thyroid binding protein and intranuclear receptor activity changes, and cytokine effects. In our study, 42.3% of the patients were identified as having NTIS, consistent with previous reports (29). Although the proportion of NTIS in the PICS and non-PICS groups was not significantly different, the T3 range at baseline was found to be an independent risk factor for PICS occurrence. Despite only free TH having biological activity, this research not only focused on the physiological role of THs but also explored the effects of inflammation, immune status, and energy metabolism on THs in sepsis. Therefore, although FT3 was not measured, the phenomena observed through T3 may also be valuable in future clinical research and treatments. In addition, we observed a difference in T4 values between the PICS and non-PICS groups, but no difference in FT4 levels. This may be due to the fact that there are changes in thyroid-binding protein levels and binding capacity in critical illness, causing the free TH level to not reflect the total level.

Tissue hypoxia in sepsis could affect the metabolism of THs, whereas supplementing THs may have favorable effects on microcirculation, mitochondria, inflammation, and immunity (30–32). Increasing evidence of an interaction between THs and inflammation, the immune system, and catabolism has been confirmed in recent years. Ilera et al. (33) found that T3, T3/T4, FT3, and FT3/FT4 levels were negatively correlated with ferritin, fibrinogen, erythrocyte sedimentation rate, CRP, lactate dehydrogenase, and D-dimer levels. Lui et al. (34) also discovered that after adjusting for some relevant factors, there was still an inverse association between CRP and FT3 levels. These results may be due to the release of cytokines (such as interleukin-1, interleukin-6, and tumor necrosis factor alpha) induced by inflammation during sepsis, which alters the activity of deiodinase and disrupts the balance between D2 and D3 deiodinase enzymes. The human body often regulates THs through three types of deiodinases and thyroid hormone receptor levels, thus changes in deiodinase activity in this situation inhibit the peripheral conversion of T4 to T3, leading to a decrease in T3 (35). A prolonged state of immunosuppression, which is one of the major components of PICS, is also a vital feature of impaired immunity in patients with severe systemic infections. Although the underlying mechanism of bidirectional correlation between the HPT axis and immune function has not yet been completely elucidated, previous *in vitro* and animal studies have suggested that THs may mediate lymphocyte proliferation and differentiation through the NF-κB pathway, protein kinase C signaling, and β-adrenergic receptor expression (14, 15). Often, when the body is in hyperthyroidism, the immune system is activated, while when patients are in hypothyroidism, the immune response is reduced. Moreover, lymphopenia, as a common clinical manifestation of severe infection, often reflects disease severity, leading to increased susceptibility to secondary and opportunistic infections (36), which can result in thyroid dysfunction. Grondman et al. (37) found that for patients with bacterial sepsis, T3 was significantly correlated with lymphocyte counts. Compared to non-lymphogenic patients, patients in severe lymphopenia had lower concentrations of T3, which was similar to our findings. THs, particularly T3, are also involved in controlling metabolic rate, appetite, sympathetic activity, and glucose and lipid metabolism (38). In addition, THs are crucial for skeletal muscle function because there are many genes regulated by T3 in muscles (39, 40), and catabolism caused by high levels of THs may result in muscle wasting. A previous study reported that in the prolonged critical illness phase, the level of T3 were negatively associated with the level of muscle protein degradation markers (11). These underlying mechanisms explain, in part, our findings that T3 was identified as an independent risk factor for PICS occurrence, and might be a good predictor for it. As similar results were not observed in the subgroup with initial thyroid disease, we postulate that over 90% of the initial thyroid disorders in our study were hypothyroidism and that these patients may have had long-term low TH levels or may have been treated with thyroid hormone supplements, which affect the regulation of the HPT axis in critical illness.

There is no consensus regarding the TH cut-off value in predicting outcomes for patients with critical illness. In a study of 100 patients that examined the association between THs and prognosis, the normal lower limit of T3 was 67 ng/dl (41). Chuang et al. (42) found that the T3 cut-off value for all-cause mortality was 52.3ng/dl in severely ill patients with acute heart failure. In our study, the lowest incidence of PICS was observed in patients with T3 in the mild deficiency range (60ng/dl-80ng/dl), and the incidence significantly increased in the SD-T3 group. Many studies have concluded that lower TH levels are accompanied by higher inflammation levels and suppressed immune function, causing patients to progress to PICS. Therefore, we speculated that during the prolonged critical illness phase, a mild deficiency in T3 could reduce the body’s catabolic metabolism, but the TH levels cannot be too low, as they are necessary to ensure immunity to decrease the incidence of PICS. Thus, a mild TH deficiency may be the optimal value for a decreased incidence of PICS, and TH supplementation in patients with a severe TH deficiency could be a viable treatment goal.

Although we concluded that a T3 level less than 60ng/dl was an independent risk factor for PICS occurrence, the influence of another important endocrine hormone, glucocorticoids, cannot be ignored. The activation of the hypothalamic-pituitary-adrenal (HPA) axis is one of the primary responses to sepsis, and for patients with septic shock, glucocorticoids are often used if their hemodynamic stability cannot be recovered after adequate fluid resuscitation and vasoactive drugs. Cortisol has been widely proven to have significant effects on metabolic and immunological homeostases. Therefore, although few studies have confirmed an association between glucocorticoids and PICS, we believe that blood glucocorticoid levels may have an impact on the occurrence of PICS. Moreover, in a long-term critical condition, circulating cortisol could inhibit the secretion of adrenocorticotropic hormone and cortisol due to the negative feedback (43), which may also inhibit the HPT axis, causing a reduction in serum TSH, T4, and T3 levels, and subsequently affecting PICS incidence. In our study, the PICS and non-PICS groups had similar initial cortisol levels. Considering that most of T3 derives from the deiodination of T4, and the half-life of T4 in plasma is 7 d, we speculate that the inhibitory effects on the hypothalamus and pituitary caused by corticosteroids had no obvious impact on baseline TH levels in this study. In addition, only 24.5% of patients undergoing corticosteroid treatment used dexamethasone, the remaining patients used hydrocortisone or methylprednisolone, which are short- or medium-acting corticosteroids that have a weak inhibitory effect on the HPA axis. In the future, with an increased sample size, we can further analyze the impact of THs on the occurrence of PICS in a subgroup without glucocorticoid treatment.

Our study has several limitations. First, this was a single-center study and the results might be affected by characteristics such as race, region, and hospital. Second, the MIMIC-IV database contains patient data from between 2008 and 2019. The treatment strategies for sepsis may have changed during this time, which is a confounding factor that could have affected our results. Third, 14,176 patients were excluded due to a lack of TH measurement. Clinically, THs are measured when there is a suspicion of thyroid disease, thus, there may be bias in the enrolled population, limiting the representativeness of these patients and the generalizability of the conclusions. However, considering that the proportion of NTIS in our study was similar to previous research and that the findings observed in the subgroup without thyroid disease were the same as those in the whole cohort, we speculate that the conclusions of this study still have some reference value for patients with sepsis. Finally, the development of PICS cannot be solely explained by baseline characteristics and treatments after admission, such as TH supplementation, were not considered in this study. The dynamic variations in THs during the hospital stay were also not explored. Further prospective studies are needed to explore these factors.

## Conclusions

In conclusion, this study found that a severe T3 deficiency in patients with sepsis was significantly associated with higher PICS incidence, and maintaining T3 within the mild deficiency range (60-80ng/dl) may be optimal. A combination of parameters can be used as a predictor of PICS in subgroups without initial thyroid disease. Accordingly, this study could contribute to the building of a predictive model for PICS and might contribute to provide a TH supplementation treatment goal in patients with a severe deficiency.

## Data Availability

The original contributions presented in the study are included in the article/**Supplementary Material**, further inquiries can be directed to the corresponding author/s.
