# Supplementary material for: Impact of thyroid hormones on predicting the occurrence of persistent inflammation, immunosuppression, and catabolism syndrome in patients with sepsis
Source: Front Endocrinol (Lausanne). 2024 Oct 16;15:1417846. doi: 10.3389/fendo.2024.1417846 (PMC11521835; doi:10.3389/fendo.2024.1417846)
Supplement: Supplementary file 1 [file Table1.docx]

Supplementary Appendix

**Impact of thyroid hormone on predicting the occurrence of persistent inflammation, immunosuppression and catabolism syndrome in patients with sepsis**

**Table S1:** Outcomes in PICS vs non-PICS class

| **Outcomes** | **PICS (n=205)** | **Non-PICS (n=671)** | **P** |
| --- | --- | --- | --- |
| **ICU length of stay (days)** | 11.4 (6.2,18.1) | 4.6 (2.9,8.0) | ＜0.001 |
| **Hospital length of stay (days)** | 26.2 (18.1,36.8) | 11.1 (7.6,15.8) | ＜0.001 |
| **ICU mortality, n (%)** | 18 (8.8) | 16 (2.4) | ＜0.001 |
| **Hospital mortality, n (%)** | 40 (19.5) | 34 (5.1) | ＜0.001 |
| **Mortality 28 days, n (%)** | 30 (14.6) | 36 (5.4) | ＜0.001 |
| **Discharge destination** |  |  | ＜0.001 |
| Home | 3 (1.5) | 104 (15.5) |  |
| Died | 40 (19.5) | 35 (5.2) |  |
| Other | 162 (79) | 532 (79.3) |  |

Data are presented as mean ± SD or median (interquartile range) for skewed variables or proportions for categorical variables.

PICS, persistent inflammation, immunosuppression and catabolism syndrome.

**Table S2:** Comparisons of PICS incidence between patients with mild and severe thyroid hormone deficiency

| **Variable** | **All** | | **Non-thyroid disease** | | **Thyroid disease** | |
| --- | --- | --- | --- | --- | --- | --- |
|  | **PICS (%)** | **P** | **PICS (%)** | **P** | **PICS (%)** | **P** |
| **T3 (ng/dl)** |  |  |  |  |  |  |
| MD-T3 (60-80) | 13 (17.8) | 0.012 | 9 (18.8) | 0.003 | 4 (16.0) | 0.321 |
| SD-T3 (＜60) | 45 (34.4) |  | 25 (47.2) |  | 20 (25.6) |  |
| **T4 (ug/dl)** |  |  |  |  |  |  |
| MD-T4 (5.5-6.8) | 17 (14.5) | ＜0.001 | 11 (15.7) | 0.001 | 6 (12.8) | 0.033 |
| SD-T4 (＜5.5) | 74 (34.1) |  | 46 (38.3) |  | 28 (28.9) |  |

Data are presented as number (percentage).

PICS, persistent inflammation, immunosuppression and catabolism syndrome; MD-T3, mild deficiency of T3; SD-T3, severe deficiency of T3; MD-T4, mild deficiency of T4; SD-T4, severe deficiency of T4.

**Table S3:** Univariate and multivariate logistic regression analysis of PICS in subgroup without thyroid disease

| **Variable** | **Univariate logistic regression analysis** | | **Multivariate logistic regression analysis** | |
| --- | --- | --- | --- | --- |
|  | **Odds ratio (95% CI)** | **P** | **Odds ratio (95% CI)** | **P** |
| Female gender | 0.945 (0.637-1.403) | 0.781 |  |  |
| Age | 1.004 (0.992-1.016) | 0.495 |  |  |
| BMI | 0.975 (0.944-1.006) | 0.114 |  |  |
| SOFA score | 1.184 (1.122-1.249) | ＜0.001 | 1.652 (1.039-2.627) | 0.034 |
| SAPS II score | 1.032 (1.018-1.046) | ＜0.001 | 0.931 (0.851-1.019) | 0.122 |
| Charlson comorbidity score | 1.094 (1.022-1.171) | 0.010 | 1.252 (0.896-1.749) | 0.188 |
| SD-T3 | 3.869 (1.568-9.549) | 0.003 | 15.508 (1.664-144.541) | 0.016 |
| T4 | 0.825 (0.709-0.959) | 0.012 | 0.909 (0.504-1.638) | 0.751 |
| WBC | 1.009 (0.983-1.036) | 0.506 |  |  |
| CRP | 1.007 (0.999-1.014) | 0.071 |  |  |
| Hemoglobin | 0.910 (0.834-0.994) | 0.035 | 1.410 (0.899-2.212) | 0.135 |
| Platelets | 0.997 (0.994-0.999) | 0.002 | 1.008 (0.996-1.019) | 0.191 |
| Lactate | 1.144 (1.059-1.236) | 0.001 | 1.129 (0.921-1.383) | 0.330 |
| AST | 1.000 (1.000-1.000) | 0.019 | 1.001 (0.999-1.002) | 0.566 |
| Creatinine | 1.000 (0.999-1.001) | 0.616 |  |  |
| Immunocompromised disease | 1.473 (0.885-2.450) | 0.136 |  |  |

CI, confidence interval; BMI, body mass index; SOFA, Sequential Organ Failure Assessment; SAPS II, Simplified Acute Physiology Score II; SD-T3, severe deficiency of T3; WBC, white blood cell; CRP, C-reactive protein; AST, aspartate transaminase.
